# Hijacking Self‐Assembly to Establish Intracellular Functional Nanoparticles

**DOI:** 10.1002/advs.202203027

**Published:** 2022-09-08

**Authors:** Yang Liu, Yuchen Wang, Chao Wang, Tiejun Dong, Haiheng Xu, Yunfei Guo, Xiaozhi Zhao, Yiqiao Hu, Jinhui Wu

**Affiliations:** ^1^ State Key Laboratory of Pharmaceutical Biotechnology Medical School and School of Life Sciences Nanjing University Nanjing 210093 China; ^2^ Department of Andrology Drum Tower hospital Medical School of Nanjing University Nanjing 210093 China; ^3^ Jiangsu Key Laboratory for Nano Technology Nanjing University Nanjing 210093 China; ^4^ Chemistry and Biomedicine Innovation Center Nanjing University Nanjing 210093 China

**Keywords:** efferocytosis of erythrocytes, endogenous metabolites, intracellular self‐assembly, nanomedicine targeting, vascular‐disrupting agent

## Abstract

The targeted transport of nanomedicines is often impeded by various biological events in the body. Viruses can hijack host cells and utilize intracellular transcription and translation biological events to achieve their replication. Inspired by this, a strategy to hijack endogenous products of biological events to assemble into intracellular functional nanoparticles is established. It has been shown that, following tumor vessel destruction therapy, injected cell permeable small molecule drugs bisphosphonate can hijack the hemorrhagic product iron and self‐assemble into peroxidase‐like nanoparticles within tumor‐infiltrating macrophages. Unlike free drugs, the generated intercellular nanoparticles can specifically stress mitochondria, resulting in immune activation of macrophages in vitro and polarizing tumor‐associated macrophages (TAMs) from immunosuppressive to tumoricidal and increasing the recruitment of T cells deep within tumor. The hijacking self‐assembly strategy significantly inhibits tumor growth compared with the treatment of vascular‐disrupting agents alone. Using bisphosphonate to hijack the metabolite associated with hemorrhage, iron, to fabricate functional nanoparticles within specific cells, which may open up new nanotechnology for drug delivery and small molecular drug development.

## Graphical abstract

Systemically injected small molecule agents bisphosphonate react with endogenous ferrous iron, the product of erythrocyte degradation, and finally self‐assembly into functional nanoparticles to regulate the behavior of cells. This virus‐inspired hijacking self‐assembly strategy brings new clinically feasible nanotechnology for the cell targeting and action mode of small molecule agents.

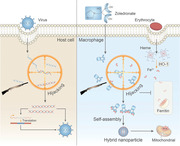

## 1 Introduction

Various types of biological events render the inability of nanomedicines to efficiently perform targeting functions. The efficiency of passive targeting strategies based on the enhanced permeability and retention (EPR) effect to reach the tumor site after systemic administration was disappointing^[^ ^1^ ^]^ and most of them were engulfed by phagocytes and accumulated in the liver and kidney.^[^ ^2^ ^]^ Besides, the active targeting strategy of surface‐modified ligands cannot work effectively because of the adsorption of a large number of endogenous proteins.^[^ ^3^ ^]^ To overcome the negative effects of massive biological events, nanoparticles are constantly undergoing more complex modifications,^[^ ^4^ ^]^ which may result in more unanticipated biological interactions.

**Figure 1:**
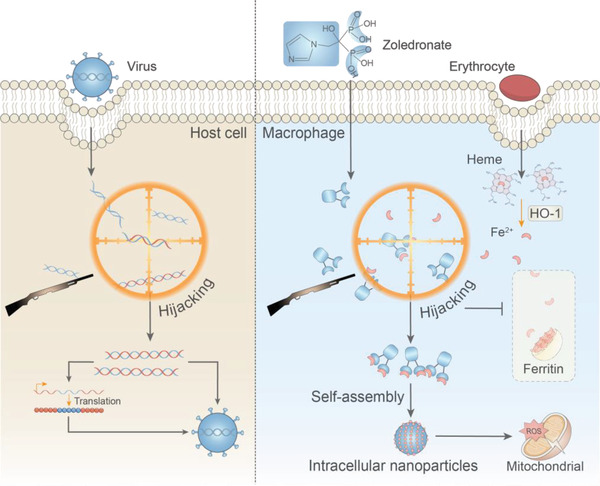
Scheme of bisphosphonate hijacks erythrocytes derived iron and assembles into Intracellular Nanoparticles to stress the mitochondrial. A virus‐inspired hijacking self‐assembly strategy. Small molecule drug bisphosphonate react with endogenous ferrous iron, the product of erythrocytes degradation, to form functional nanoparticles in situ.

Compared with inanimate nanoparticles, many viruses break through the biological defense of the host with simple nanostructures and easily replicate themselves in host cells. After entering the cell, the viruses hijack the host genome and realize their own replication by using biological events such as transcription and translation of the host cell.^[^ ^5^ ^]^ Inspired by this, we proposed a hijacking self‐assembly strategy (**Figure** **1**). That is, based on the biological events in the organism, small molecular drugs that have been listed in the clinic are systematically given as reactants, so that they can react with the products of specific biological events, and finally form functional nanoparticles to regulate the behavior of cells.

In this study, we selected a physiological process frequently occurring in vivo, efferocytosis of erythrocytes. Extravasated erythrocytes are phagocytosed by macrophages and free iron is released under the action of heme oxygenases‐1 (HO‐1). Generally, to circumvent free radicals, Fe^2+^ produced by degraded erythrocytes is oxidized and encapsulated for storage by ferritin and pumped out of the macrophages by ferroportin 1.^[^ ^6^, ^7^ ^]^ Similar to viruses hijacking the host genome, bisphosphate,^[^ ^8^ ^]^ as a class of clinically used drugs, can hijack Fe^2+^ released from erythrocytes in a coordinated manner and self‐assemble into peroxidase‐like nanoparticles, which can catalyze hydrogen peroxide, continuously stress macrophage mitochondria, activate and remodel the antitumor immune response of TAMs to inhibit tumor growth. Since little free iron is present in normal tissues, bisphosphonate do not form nanoparticles when circulating in normal tissues, effectively circumventing off‐target toxicity. This hijacking self‐assembly mimics the process of virus hijacking of host cells and targeted generation of functional nanoparticles using biological events.

## 2 Results and Discussion

### 2.1 Erythrocytes Undergo Efferocytosis in Tumors Induced by Vascular‐Disrupting Treatment

**Figure 2:**
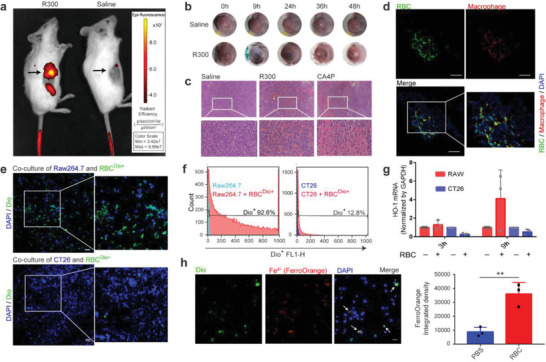
TAMs degrade infiltrated erythrocytes after vascular‐disrupting treatment. a) DiR stained erythrocytes accumulated in mouse tumors after anti‐platelet antibody (R300) treatment. b) Tumors in mice taken at different time points after R300 treatment. c) H & E staining of tumor tissue sections from mice after treatment with different drugs. d) The colocalization of RBC (green) with macrophages (red) of mice tumor tissue sections after hemorrhage induction by R300. Scale bars, 100 µm. e) The colocalization of erythrocytes (green) with macrophages (blue) other than CT26 (blue), which were coincubation in vitro. Scale bars, 100 µm. f) DiO stained erythrocytes coincubating with tumor cells or macrophages. g) qPCR detected the expression changes of HO‐1 of macrophages and CT26 after erythrocytes treatment (*n* = 3 per group); h) Intracellular Fe^2+^ contents of macrophages treated with DiO stained erythrocytes (*n* = 3 per group). Scale bars, 100 µm. The RBC represent erythrocyte. Data are shown as the mean ± SD, *p < 0.05, **p < 0.01, ***p < 0.001, and ****p < 0.0001. The data were analyzed by Student's *t*‐test (h).

A common feature shared by almost all solid tumors is the large number of structurally abnormal vascular networks.^[^ ^9^ ^]^ Our previous work found that tumor vascular homeostasis can be selectively destroyed.^[^ ^9^, ^10^ ^]^ Here, we transfused erythrocytes stained with DiR into mice. When using an anti‐platelet antibody (R300) to destroy the homeostasis of tumor vascular endothelial cells,^[^ ^11^ ^]^ we found that the original nontumor targeting erythrocytes could infiltrate into the tumor in large numbers (**Figure** **2**a). The blackness of hemorrhage in mouse tumors disappeared within 24 h (Figure 2b). Many erythrocytes could be seen in the tumor tissue section after R300 administration (Figure 2c). This means that certain cells in the tumor cleared the tumor infiltrated erythrocytes during this time. To find out which cells cleared tumor infiltrated erythrocytes, isolated erythrocytes were stained with green fluorescent membrane dye in vitro and then intravenously injected into mice. After platelet inhibition, immunofluorescence staining of tumor sections showed that the green fluorescence of erythrocytes and the F4/80 marker of macrophages were highly colocalized, indicating the possibility of phagocytosis by macrophages (Figure 2d).

To further clarify the phagocytic relationship, we cocultured the stained erythrocytes with Raw264.7 macrophages and CT26 tumor cells. After washing away the suspended erythrocytes, the macrophages and tumor cells adhering to the bottom of the plate were analyzed by flow cytometry and confocal microscopy. Tumor cells rarely phagocytized erythrocytes, but macrophages phagocytized a large number of erythrocytes (Figure 2e,f). Heme oxygenase is required for free iron production after hemoglobin engulfment. We used qPCR to show that HO‐1 expression was significantly upregulated after coincubation of macrophages and erythrocytes, but not tumor cells (Figure 2g). Intracellular Fe^2+^ can be detected by a commercial FerroOrange fluorescent probe. Compared with normal macrophages, macrophages that engulf erythrocytes showed stronger fluorescence (Figure 2h).

Although tumor cells have active pinocytosis, we speculated that erythrocytes are too large to be engulfed as their diameters are approximately 8 µm. To verify this, we broke the stained erythrocytes into nanofragments and repeated the coculture experiment. The results indicated that tumor cells could phagocytize the nanosized erythrocyte fragments, with no significant difference compared to macrophages (Figure S1, Supporting Information). Many studies have found that erythrocytes infiltrating tissues from blood vessels will quickly enter apoptosis due to changes in osmotic pressure, energy depletion, and coagulation reactions.^[^ ^12^ ^]^ We used phorbol myristate acetate (PMA) to induce phosphatidylserine eversion of the erythrocyte membrane (Figure S2, Supporting Information), and an antibody to block CD47 (Figure S3, Supporting Information), the phagocytosis of erythrocytes by macrophages was significantly enhanced.

Iron is an essential element for cell metabolism, and systemic administration of iron is intercepted by tumor cells overexpressing transferrin receptors and promotes tumor cell growth (Figure S4a, Supporting Information). We found that unlike free iron, erythrocytes do not promote tumor cell growth and can also develop weak oxidative toxicity (Figure S4b,c, Supporting Information).

Collectively, erythrocytes circulating in blood vessels can accumulate in tumor tissue by selectively breaking vascular endothelial homeostasis. The clearance of erythrocytes in tumors is mainly performed by macrophages, whereas tumor cells are hardly involved in this process. TAMs can rapidly release Fe^2+^ from erythrocytes by upregulating HO‐1. Therefore, by inducing tumor hemorrhage, macrophages in the tumor can be used to digest endogenous erythrocytes and produce a large amount of free Fe^2+^.^[^ ^6^ ^]^

### 2.2 Bisphosphonates Hijack Erythrocyte Derived Fe^2+^ for Assembly into Nanoparticles within Macrophage

Free Fe^2+^ usually exists for a short time in cells and ferritin encapsulates it after oxidization to Fe^3+^.^[^ ^13^ ^]^ Inspired by the virus hijacking host cells to produce new virus particles, we speculated that intracellular free Fe^2+^ can self‐assemble into nanoparticles with suitable small molecule drugs with high cell penetration.

**Figure 3:**
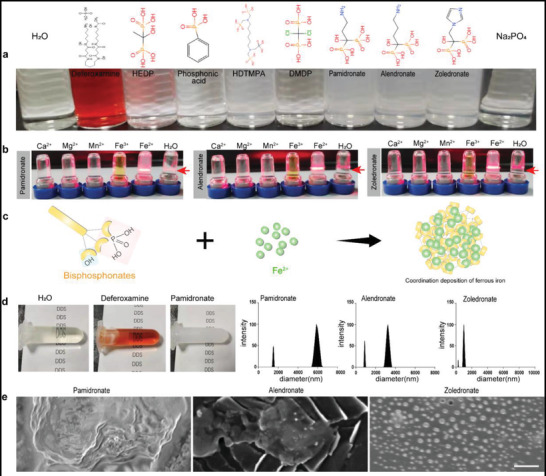
Bisphosphonate selectively coordinate with Fe^2+^ to produce nanoparticles. a) Photographs of self‐assembly of coordination reactions with different ligands and Fe^2+^. b) Self‐assembly after coordination with different metal ions with three kinds of bisphosphonate. c) Scheme of coordination self‐assembly of bisphosphonate and Fe^2+^. d) Results of particle size detection after coordination with different bisphosphonate and Fe^2+^. e) Scanning electron microscope photographs of self‐assembled nanoparticles reacted with different bisphosphonates and Fe^2+^. Scale bars, 200 nm. The BPS represents bisphosphonate.

Deferoxamine (DMF), which coordinates iron, has been applied in the clinic. However, it produces coordination compounds with better dissolution performance and cannot achieve the goal of precipitating Fe^2+^ (**Figure** **3**a). Analyzing the precipitation dissolution equilibrium constant of iron salts, it can be seen that the phosphate group has the worst solubility. Therefore, we selected phosphate‐containing drug molecules by carrying out the reaction in an aqueous solution and found that bisphosphonate with a side chain as organic carbon chains can deposit Fe^2+^ quickly, while inorganic sodium phosphate and bisphosphonate with side chains can deposit Fe^2+^ as chloride ions with low precipitation efficiency and slow speed (Figure 3a). This is due to the hydrophobic environment created by the organic side chains of bisphosphonate, allowing more rapid assembly of the ligands together. Furthermore, we found that this coordinated self‐assembly phenomenon is selective, and at the same molarity, only Fe^2+^ can form precipitates with bisphosphonate (Figure 3b). More interestingly, zoledronate whose side chain is an imidazole ring can produce a large amount of uniformly dispersed nanoparticles with diameters less than 1000 nm (Figures 3d,e, S12, Supporting Information). This may be due to the larger cavity in the imidazole ring compared to the chain‐like side chains, and the steric hindrance makes it possible to disperse each other between the products (Figure 3c).

**Figure 4:**
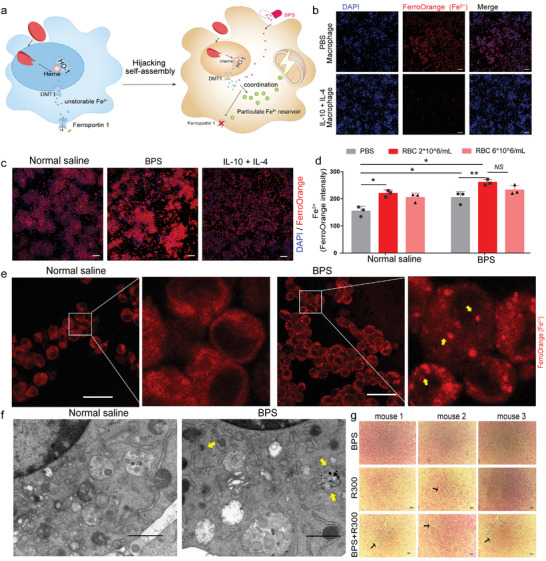
Bisphosphonates hijack erythrocyte‐Fe^2+^ for assembly into nanoparticles within macrophages. a) Scheme of bisphosphonate hijacks erythrocytes derived Fe^2+^ and assembly into nanoparticles within macrophages. b) Intracellular Fe^2+^ of macrophages induced by IL‐4 as well as IL‐10 using fluorescent probes. Scale bars, 100 µm. c, d) Intracellular Fe^2+^ of macrophages treated with erythrocytes and bisphosphonates (n = 3 per group). Scale bars, 100 µm. e) Detection of intracellular Fe^2+^ nanoparticles of macrophages treated with bisphosphonate by ferrous iron fluorescent probe. Scale bars, 12 µm. f) Transmission electron microscope image of macrophage treated by bisphosphonate. Scale bars, 1 µm. g) Photographs of Prussian blue staining of the CT26 tumor bearing mice in the treatment at Day16 (n = 3 per group). Scale bars, 50 µm. Data are shown as the mean ± SD. ^NS^ *p* > 0.05, *p < 0.05, **p < 0.01, ***p < 0.001, and ****p < 0.0001. The data were analyzed by one‐way ANOVA (d).

TAMs overexpress phagocytosis‐related receptors, including CD163, which can recognize hemoglobin. However, unlike undifferentiated macrophages, most TAMs overexpress iron metabolism‐associated proteins, such as transferrin and ferroportin 1 (**Figures** **4**a, S4a, Supporting Information). We used interleukin‐4 (IL‐4) and IL‐10 treatment to induce anti‐inflammatory macrophages and found that their intracellular iron concentration was significantly reduced (Figure 4b). Consistent with the results of extracellular iron precipitate screening, bisphosphonate‐treated macrophages deposited more Fe^2+^ after phagocytosis of erythrocytes than normal saline‐treated macrophages (Figure 4c,d). Flow cytometry results showed that bisphosphonate did not affect the phagocytosis of erythrocytes by macrophages (Figure S5, Supporting Information). In *ex vivo* observation, when erythrocytes were cocultured with bisphosphonate‐treated macrophages, Fe^2+^‐Bisphosphonate Nanoparticles could be clearly observed by using fluorescence imaging (Figure 4e) and transmission electron microscopy (Figure 4f), which indicated that the self‐assembly could occur in cells. We used R300 to destroy the homeostasis of mouse tumor vascular endothelial cells. Bisphosphonate does not affect the survival of macrophages in the tumor (Figure S6, Supporting Information) and normal cell (Figure S13, Supporting Information). Prussian blue staining showed more iron particle deposition in the tumor when treated with the combination of R300 with bisphosphonate (Figure 4g), suggesting that the hijacking self‐assembly can proceed in vivo.

Intracellular accumulation of small molecules can be addressed by self‐assembly into nanoparticles and holds great potential for applications in molecular imaging.^[^ ^14^, ^15^, ^16^ ^]^ We showed that cell‐penetrable small molecule drugs can effectively react with endogenous metabolites and self‐assemble to form nanoparticles. Normal tissues without targeted biological events will not undergo self‐assembly, which makes the hijacking self‐assembly highly targeted and provides a new idea of drug targeting.

### 2.3 Intracellular Nanoparticles Specifically Stress Mitochondria

**Figure 5:**
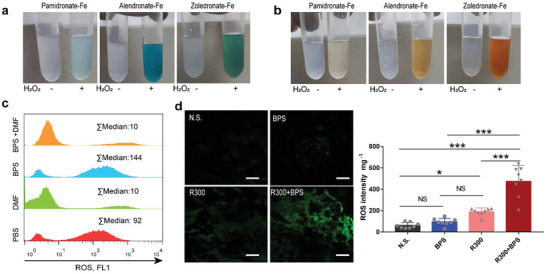
Peroxidase activity of Fe^2+^‐Bisphosphonate Nanoparticles. a,b) Peroxidase activity detection of extracellular Fe^2+^‐Bisphosphonate Nanoparticles catalysts. The chromogen used for (a) was TMB: Tetramethylbenzidine and (b) was DAB: Diaminobenzidine. c) ROS detection of macrophages engulfment of erythrocytes following with zoledronate (BPS) or deferoxamine (DMF) by flow cytometric. d) ROS generation of the CT26 tumor bearing mice in the treatment of bisphosphonates and R300 at Day 3 (n = 8 per group). Scale bars, 100 µm. The RBC represents erythrocyte. Data are shown as the mean ± SD. ^NS^p > 0.05, *p < 0.05, **p < 0.01, ***p < 0.001, and ****p < 0.0001. One‐way ANOVA for group comparisons (d).

Among the already marketed nanomedicines, all systemically injected nanomedicines function by releasing free drugs, which makes nanotechnology replaceable among these nanomedicines. Nanoparticles have many special properties. For example, it is found that iron nanoparticles (nanoFe_3_O_4_) have catalytic properties.^[^ ^17^ ^]^ However, the inflammation‐related mechanism of iron nanoparticle‐activated macrophages is not clear.^[^ ^18^ ^]^ Similar to the peroxidase activity of nanoFe_3_O_4_,^[^ ^15^ ^]^ we found that the self‐assembled nanoparticles could also catalyze hydrogen peroxide to induce tetramethylbenzidine (TMB) and diaminobenzidine (DAB) chromogenic oxidation (**Figure** **5**a,b). Reactive oxygen species (ROS) in macrophages were stained by DCFH‐DA and were increased by bisphosphonate and decreased by DMF (Figure 5c). Tumor tissue sections also showed that tumors in the combination group produced more ROS (Figure 5d).

**Figure 6:**
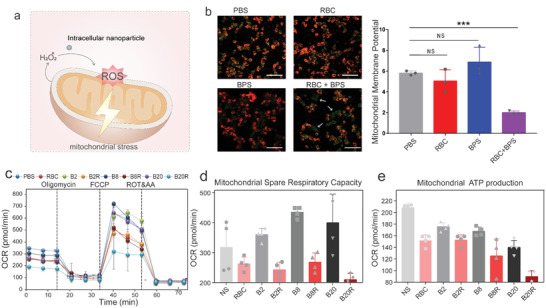
Intracellular Nanoparticles stress mitochondria. a) Hypothetical mechanism diagram of ROS generation by Intracellular Nanoparticles. b) Detection of mitochondrial membrane potential of macrophages treated with erythrocytes and bisphosphonate by JC‐10 (n = 3 per group). Scale bars, 100 µm. c) Intracellular Nanoparticles inhibit cellular oxygen consumption in macrophages. At 6 h after incubation with bisphosphonate (2, 8, 20 µM, abbreviated as Z) alone and combine with erythrocytes (5 ×10^6^ per 1 mL, abbreviated as R), the cellular OCR was measured by Seahorse analyzer. Oligomycin (1 µM) was introduced after 20 min, FCCP (1 µM) was introduced after 35 min and rotenone/antimycin A (0.5 µM) was introduced after 60 min (*n* = 4 per group). d, e) Cellular mitochondrial spare respiratory capacity (d) and ATP level (e) of macrophage with erythrocytes (5 ×10^6^ per 1 mL) and bisphosphonate (2, 8, 20 µM) (n = 4 per group). The RBC and BPS abbreviations represent erythrocytes and zoledronate, respectively. Data are shown as the mean ± SD. ^NS^ *p* > 0.05, *p < 0.05, **p < 0.01, ***p < 0.001, and ****p < 0.0001. The data were analyzed by one‐way ANOVA (b).

Mitochondria are the main sites of hydrogen peroxide production. We used JC‐10 to examine mitochondrial membrane potential and found significant damage to mitochondria in macrophages treated simultaneously with bisphosphonate and erythrocytes (**Figure** **6**a,b). For mitochondrial function, we used the SeaHorse assay to detect macrophages treated with bisphosphonate combined with erythrocytes and found that the combination group had a significant decrease in oxygen consumption and in the mitochondrial spare respiratory capacity as well as ATP production (Figure 6c,d,e).

Our results indicated that the assembled Intracellular Nanoparticles may not have a protein shell and can freely contact hydrogen peroxide in the cytoplasm, resulting in oxidation pressure on the mitochondria of macrophages, which cannot be achieved by free bisphosphonate. We proposed that small molecule drugs can function by assembling into nanoparticles. This unbound way of working puts forward new nanotechnology for small molecule drugs and has important applications in the regulation of protein function in cells.

### 2.4 Intracellular Nanoparticles Trained a Strong Pro‐Inflammatory Macrophage In Vitro and Reverse the Immunosuppression of TAMs In Situ

**Figure 7:**
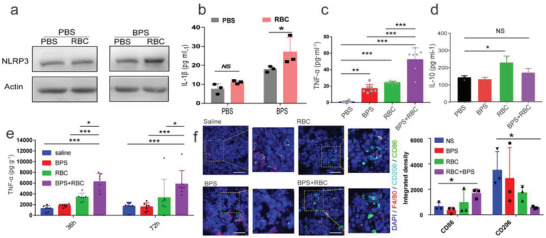
Intracellular Nanoparticles trained a strong pro‐inflammatory macrophage in vitro and reverse the immunosuppression of TAMs in situ. a) Western blot analysis of macrophages NLRP3 expression treated with the combination of bisphosphonate and erythrocytes (5 ×10^6^ per 1 mL). b) Results of IL‐1*β* expression by macrophages after phagocytosis of erythrocytes (*n* = 3 per group). c, d) The expression changes of TNF‐*α* (*n* = 7 per group) and IL‐10 (*n* = 3 per group) after macrophages with erythrocytes and bisphosphonate treatment alone or in combination treatment. e) TNF‐*α* in tumors from CT26 tumor bearing mice post various treatments (*n* = 7 per group). f) Immunofluorescence detection of tumor tissues (left) and quantification of the immunofluorescence photographs (right). Tumor tissue was collected and stained 72 hours after injection treatment (n = 3 per group). Scale bars, 50 µm. Data are representative or pooled and are expressed as Mean ± SD. The RBC and BPS abbreviations represent erythrocytes and zoledronate, respectively. ^NS^ *p* > 0.05, *p < 0.05, **p < 0.01, ***p < 0.001, and ****p < 0.0001. The data were analyzed by one‐way ANOVA (c, d) or two‐way ANOVA (e, f).

Mitochondrial stress can bring the activation of immune cells. To examine the biological effects of Intracellular Nanoparticles, we cocultured erythrocytes and bisphosphonate‐treated macrophages, and the results showed that nucleotide‐binding oligomerization domain, leucine‐rich repeat, and pyrin domain‐containing 3 (NLRP3) (**Figure** **7**a) and IL‐1*β* (Figure 7b) expression was significantly upregulated in macrophages. Bisphosphonate also significantly upregulated tumor necrosis factor‐*α* (TNF‐*α*) expression (Figure 7c) and inhibited IL‐10 upregulation (Figure 7d) after erythrocytes treatment. We simultaneously treated macrophages with guanosine 5ʹ‐diphosphate (gdp),^[^ ^19^ ^]^ an iron mobilizer, and the inhibitory effect of bisphosphonate on IL‐10 expression disappeared (Figure S7, Supporting Information). On surface marker detection, we found that bisphosphonate could upregulate CD86 expression of macrophage immune costimulatory molecules in a dose‐dependent manner, while the upregulation effect of bisphosphonate was attenuated when DMF was used simultaneously, and the inflammation inhibition related marker CD206 was also upregulated (Figure S8, Supporting Information). These results suggest that the Intracellular Nanoparticles generated through hijacking self‐assembly can generate proinflammatory macrophages.

The high plasticity of macrophages allows TAMs to be re‐regulated into a pro‐inflammatory phenotype, which recruits Th1 cells and promotes associated inflammatory responses.^[^ ^20^ ^]^ However, the polarization of macrophages is an ongoing process, the immunosuppressive microenvironment that the tumor has already established is likely to reverse the regulatory effects, and the continuous maintenance of the regulation of macrophages is a puzzle.

We injected erythrocytes as well as bisphosphonate into the tumor site of mice at various time points to detect immune factors within the tumor and found that higher levels of TNF‐*α* arise in the tumors of mice in the combination treatment group after 36 and 72 h of injection, respectively (Figure 7e). This illustrates that macrophage inflammation can be maintained by Intracellular Nanoparticles. At 72 h after treatment, immunofluorescence of tumors revealed that more CD86‐expressing proinflammatory macrophages were present in tumors of mice receiving erythrocytes in combination with bisphosphonate treatment, while CD206‐expressing macrophages were also significantly reduced (Figure 7f). These results illustrated that Intracellular Nanoparticles could generate proinflammatory macrophages and reverse the immunosuppression of TAMs in situ.

### 2.5 Hijacking Self‐Assembly Strategy Inhibits Tumor Growth in Mice and Induces the Deep Infiltration of T Cells into the Tumor

**Figure 8:**
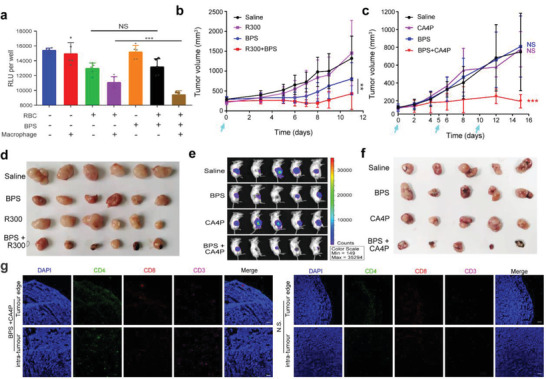
Hijacking self‐assembly strategy inhibit tumor growth in mice and induce the deep infiltration of T cells into the tumor. a) Relative luminescence unit of luciferase labeled CT26 post with macrophages, erythrocytes, as well as bisphosphonate alone or in combination (*n* = 5 per group). b) Tumor volumes of the CT26 tumor bearing mice post various treatments. R300: 2 mg^–1^ kg^–1^ anti‐platelet antibody, BPS: 2 mg^–1^ kg^–1^ zoledronate, R300+BPS: the combination of anti‐platelet antibody and zoledronate. Drugs are given on Day 0 by tail vein injection (*n* = 6 per group). c) Tumor volume of the CT26 tumor bearing mice post various treatments. CA4P: 50 mg^–1^ kg^–1^ combretastatin A4 disodium phosphate given by intraperitoneal injection, BPS: 1 mg^–1^ kg^–1^ zoledronate given by tail vein injection, CA4P+BPS: the combination of combretastatin A4 disodium phosphate and zoledronate. Drugs are given on Days 0, 5, and 10 (*n* = 5 per group). d,f) Photographs of CT26 tumors separated from mice after various treatments on Day14. e) Relative luminescence unit of luciferase labeled CT26 tumors after treatment 14 days (*n* = 5 per group). g) Fluorescent sections of intratumoral T cell infiltration in CT26 tumor bearing mice with BPS and CA4P treatment. Scale bars, 100 µm. Data are shown as the mean ± SD. ^NS^ *p* > 0.05, *p < 0.05, **p < 0.01, ***p < 0.001, and ****p < 0.0001. The data were analyzed by one‐way ANOVA (a) or two‐way analysis of ANOVA (b, c).

More tumor cytotoxicity was exhibited by hemophagocytic macrophages after the formation of Fe^2+^‐Bisphosphonate Nanoparticles. The addition of bisphosphonate to the tumor cells cocultured with erythrocytes did not decrease the viability of the tumor cells, whereas cell viability significantly decreased if macrophages were present simultaneously (**Figure** **8**a).

To explore the antitumor potential of the Intracellular Nanoparticles produced by hijacking assembly in vivo, we individually subjected two mouse models of tumor hemorrhage. After tumor hemorrhage was induced by antiplatelet antibody (R300) and vascular disrupting agents (combretastatin A‐4‐phosphate, CA4P), the mice were treated with bisphosphonate. Compared with single use, the combination of R300 or CA4P could significantly inhibit the growth of tumors (Figure 8b,c). Meanwhile, these tumors were significantly smaller in size and appeared necrotic with darkening (Figures 8d,f, and S9, Supporting Information). DAPI staining showed that the structure of the tumor became loose (Figure S10, Supporting Information). Detection using an autoluminescent approach revealed that the luminescent intensity of mouse tumors was also significantly reduced (Figure 8e). Vascular disrupting agents have passed clinical safety trials, and bisphosphonate has been used in the clinic for many years, and no significant toxicity was detected in the major organs of the mice treated in the combination group (Figure S14, Supporting Information). Our hijacking self‐assembly may be quickly transformed and applied to the clinic.

A more exciting finding is that more CD11c^+^ and CD3^+^ cells were found in tumors by flow cytometry (Figure S10, Supporting Information). Whether T cells can penetrate deep into the tumor is one of the keys to their function. To further observe the infiltration location of T cells, we performed immunofluorescence detection of tumors after the simultaneous use of vascular disruption agents and bisphosphonate treatment (Figure 8g). We cocultured murine T cells and macrophages in vitro and found that macrophages that had engulfed erythrocytes could significantly elevate granzyme B release from CD8^+^ T cells (Figure S11, Supporting Information). This finding indicated that hijacking self‐assembly strategy trained macrophages can also recruit and boost T cells to attack tumors.

## 3 Conclusion

We show that cell‐penetrable small molecule drugs can effectively react with endogenous metabolites and self‐assemble to form nanoparticles. The self‐assembled Fe^2+^‐Bisphosphonate Nanoparticles have peroxidase‐like catalytic function and can specifically stress mitochondria, which cannot be achieved by free bisphosphonate. This biomimetic synthesis strategy avoids the impact of unexpected biological events on nanoparticles and provides a new idea for nanomedicine targeting. Compared with various complex modifications of nanoparticles, hijacking self‐assembly is easy to operate and has higher clinical feasibility. This strategy also shows that small molecule drugs can function by assembling nanoparticles with endogenous substances, which puts new nanotechnology for small molecule drugs working.

## Conflict of Interest

The authors declare no conflict of interest.

## Authors Contribution

J.H.W. and Y.L. planned and coordinated the overall research; Y.L., J.H.W., and C.W. designed and performed experiments and data analysis. Y.L., J.H.W., H.H.X., Y.Q.H. Y.F.G., C.W., Y.C.W., and T.J.D. provided technical advice; Y.L., J.H.W., C.W. provided scientific and experimental advice and edited the manuscript.

## Supporting information

Supporting information

## Data Availability

The data that support the findings of this study are available from the corresponding author upon reasonable request.
