# Supplementary material for: Hijacking Self‐Assembly to Establish Intracellular Functional Nanoparticles
Source: Adv Sci (Weinh). 2022 Sep 8;9(31):2203027. doi: 10.1002/advs.202203027 (PMC9631083; doi:10.1002/advs.202203027)
Supplement: Supplementary file 1 — Supporting information [file ADVS-9-2203027-s001.pdf]

ADVANCED SCIENCE
Open Access

Supporting Information

for *Adv. Sci.*, DOI 10.1002/advs.202203027

Hijacking Self-Assembly to Establish Intracellular Functional Nanoparticles

Yang Liu, Yuchen Wang, Chao Wang, Tiejun Dong, Haiheng Xu, Yunfei Guo, Xiaozhi Zhao, Yiqiao Hu* and Jinhui Wu**

Supporting Information

Hijacking self-assembly to establish intracellular functional nanoparticles

Yang Liu, Yuchen Wang, Chao Wang, Haiheng Xu, Tiejun Dong, Yunfei Guo, Xiaozhi Zhao, Yiqiao Hu, Jinhui Wu*.*

1. Experimental Section

Reagents: Anti-platelet antibody (polyclonal anti-mouse GPIbα rat IgG) was purchased from Emfret Analytics. Anti-F4/80 antibody, anti-CD80 antibody, anti-CD3 antibody, anti-CD4 antibody, anti-CD8a antibody, anti-CD86 antibody, and anti-CD206 antibody were purchased from Biolegend. Combretastatin A4 phosphate was purchased from Shanghai Macklin Biochemical Co., Ltd. Phorbol 12-myristate 13-acetate (PMA) was purchased from Lianke Biotech Co., Ltd. InVivoMab anti-human/mouse/rat CD47 was purchased from Bio X cell. DAPI, DiO, DiI, DiR were supplied by KeyGEN BioTECH. RPMI 1640, Fetal bovine serum (FBS), Trypsin-EDTA and penicillin were purchased from Gibco (Thermo fisher, US). Mouse TNF-α and IL-10 ELISA Kit were purchased from Hangzhou Lianke Biotechnology Co., Ltd. Alendronate, pamidronate and zoledronate were purchased from Dalian Meilun Biotechnology Co., Ltd. Dimethyl sulfoxide (DMSO) and ethanol were obtained from Sinopharm Chemical Reagent Co. Calcein AM and propidium iodide (PI) were provided by Sigma-Aldrich Chemical Corporation. Cell counting kit 8 (CCK8) was supplied by Dojindo Laboratories (Japan). Lymphoprep reagent was purchased from Tianjinhaoyang Biological Manufacture Co.,Ltd. All other reagents were obtained from Sigma-Aldrich.

Cell cultures: CT26, 293 and BHK cells were obtained from the Cell Bank of Shanghai Institutes for Biological Sciences, Chinese Academy of Sciences (Shanghai, China).

Animals: BALB/c mice were purchased from Yangzhou University Medical Center (Yangzhou, China) and the weight of the mice was approximately 16-22 g. All animal tests

and experimental procedures were conducted according to the Administration Committee of Experimental Animals in Jiangsu Province and the Ethics Committee of Nanjing University.

Mice erythrocytes preparation: The blood of BALB/c male mice was collected into syringes containing 4% sodium citrate solution as anticoagulant. Human blood was obtained from the Drum Tower Hospital affiliated to Nanjing University after informed healthy human volunteers' consent, according to protocols from the Nanjing University. Generally, the blood was were prepared with Lymphoprep reagent, centrifuged at 600 g for 10 min and erythrocytes were collected into fresh tubes. The erythrocytes were further centrifuged at 200 g for 5 min to remove the lymphatic separation fluid. The mice or human erythrocytes were then resuspended in PBS for further testing.

Tumor models: BALB/c male mice were housed in groups of five or six with free access to abundant food and water. CT26 cells were cultured in RPMI-1640 medium supplemented with 10% fetal bovine serum and 1% 200 mM L-glutamine. To establish CT26 tumor-bearing mice, the hairs on the flanks of the mice was removed before they received further treatments. BALB/c mouse subcutaneous tumor models were established by subcutaneously injecting 1×10^7 CT26 cells into the lower flanks of the mice. When the tumor volume reached approximately 150 mm^3, the tumor mass was removed and cut into small pieces approximately 4 mm^3, and the pieces were subcutaneously implanted into other mice.

In vivo analysis of erythrocytes in tumors: BALB/c mice bearing subcutaneous CT26 tumors (approximately 200 mm^3) were selected. Each tumor-bearing mouse was infused with 1×10^8 DiR-stained erythrocytes. anti-platelet antibody (2 mg/kg) was administered to each mouse 1 h after injection. The tumor accumulation of erythrocytes was detected by an IVIS Lumina II instrument 12 h later.

In vivo analysis of erythrocytes efferocytosis. one hour after infusion of 1×10^8 DiO-stained erythrocytes, 2 mg/kg anti-platelet antibody was administrated to each tumor-bearing mouse to induce bleeding. Seventy-two hour later, the mice were sacrificed to collect tumors to

evaluate the intratumor immune cell infiltration. Tumors were embedded in OCT and cut into 8 μm slices, followed by staining with PE anti-F4/80 antibody and DAPI according to the manufacturer's protocols. Finally, fluorescence images of immune-fluorescent staining were obtained by confocal fluorescence microscopy (OLYMPUS FV3000).

In vitro analysis of erythrocytes efferocytosis: CT26 and Raw264.7 cells were pre-seeded in 6-well plates and incubated for 24 h. Then, 4×10^6 DiO-stained erythrocytes were added into each well and incubated for 6 h. Subsequently, the cells were washed once with PBS. The cells were then centrifuged and resuspended in 1 mL PBS. Then, the cells were analyzed by flow cytometry (FACS-Calibur, BD Corp.). Data were obtained and analyzed using the FlowJo program.

We pre-seeded CT26 and Raw264.7 cells in a glass bottom cell culture dish at a density of 2.5×10^4 cells per 1 mL and incubated them for 24h. Then 4×10^6 DiO-stained erythrocytes were added to each dish and incubated for 6 h. Subsequently, we washed the cells with PBS for 2 times and used DAPI to dye cell nuclear. Fluorescence images of cells were obtained by confocal fluorescence microscope (OLYMPUS FV3000).

Quantitative Real-Time PCR: Total RNA from cell samples were isolated using Trizol (Tiangen BioTECH) according to the manufacturer's instructions. Two micrograms of total RNA were adapted for qRT-PCR (Applied Biosystem) to generate cDNA. Quantitative Real-Time PCR was carried out using a Light Cycler 96 (Roche) and Fast Start Essential DNA master mix (2X) reagent. Cq values normalized relative to the expression of endogenous control genes using $2^{(-\Delta\Delta Cq)}$ were plotted. The upstream and downstream primer sequences for HO-1 were as follows: CAGTCCCAAACCTCGCGGT; GCTGTGCAGGTGTTGAGCC.

Peroxidase-like activity assays of aggregate catalysts: The coordination reaction was performed using an excess of bisphosphonate and the same concentration of Fe^{2+}, and the coordination precipitates were collected using centrifugation and cleaned once with pure water. The precipitates were resuspended using an equal volume, and an equal volume was taken for detection. The H_2O_2-catalytic reaction system included H_2O_2 at a consistent

concentration of 100 mM, DAB or TMB at a concentration of 10 mM. The oxidation product of TMB produces a blue signal and DAB produces a brown signal. All tubes were incubated for 5 min in the dark at room temperature and then imaged and analyzed.

Characterization of self-assembled nanoparticles: The metal salt and bisphosphonate were dissolved in distilled water, and the metal salt solution and different bisphosphonates containing phosphate groups into the transparent glass bottle respectively, wait for two minutes, and then take photos. Scanning electron microscope (SEM, JEM-2100, Japan) was used to visualize the structure of self-assembled Fe^{2+}-bisphosphonate nanoparticles. Particle size for different phosphonate were measured by Dynamic Light Scattering (DLS, Brookhaven 90 plus Zeta).

Ex vivo analysis of self-assembled nanoparticles: Macrophages were pre-seeded in cell culture dishes and incubated for 12 h, 2×10^7 fresh erythrocytes were added to each dish and incubated for 24 h. Cells were washed twice with normal saline, then treated with 1 mM zoledronate for 12 h at 37 ℃. The cells were collected by centrifugation and then observed using a transmission electron microscope (Hitachi ht7700 120K).

Detection of intracellular Fe^{2+} amount: To detect intracellular Fe^{2+}, FerroOrange (Goryo Chemical Inc., Hokkaido, Japan) was used according to the manufacturer's protocol. Cells on glass bottom cell culture dishes were washed twice with Hank's Balanced Salt Solution (HBSS) to remove the residual. Then the cells were treated with 1 mM FerroOrange with HBSS for 30 min at 37 ℃. After incubation, FerroOrange was removed by washing twice with HBSS. Fluorescence images of cells were obtained by confocal fluorescence microscope (OLYMPUS FV3000) or Microplate Reader.

Mitochondrial Membrane Potential: Mitochondrial membrane potential was assessed using Mitochondrial Membrane Potential Kit (JC-10 assay, KeyGEN BioTECH, China). JC-10 was used according to the manufacturer's protocol. Cells on glass bottom cell culture dishes were washed twice with Hank's Balanced Salt Solution (HBSS) to remove the residual and then

treated with JC-10. Fluorescence images of stained cells were obtained by confocal fluorescence microscope (OLYMPUS FV3000).

Seahorse assay: Mitochondrial function was assessed with an extracellular flux analyzer (Seahorse Bioscience). Individual wells of XF96 cell culture microplates were coated with CellTak in accordance with the manufacturer's instructions. The matrix was adsorbed overnight at 37 °C until use. Macrophages were plated and incubated overnight at 37 °C. Twenty-four hours later, different concentrations of zoledronic acid (2, 8, 20 μM) alone or combined with erythrocytes (5×10^6 per 1 mL) were added to each well and incubated for another 6 hours. During instrument calibration, the cells were washed twice with XF assay medium containing 10 mM glucose and 2 mM L-glutamine. The cells were switched to a CO_2-free (37 °C) incubator for 30 min. XF96 assay cartridges were calibrated in accordance with the manufacturer's instructions. Cellular OCRs and ECARs were measured under basal conditions and following treatment with 1 μM oligomycin, 1 μM FCCP, and 500 nM rotenone/antimycin A (XF Cell Mito Stress Kit, Seahorse Bioscience).

Immuno-activation characterization of macrophage in vitro: Fluorescence image of cells were obtained by confocal Raw264.7 were pre-seeded in glass bottom cell culture dish and incubated for 12 h. 4×10^6 or 2×10^7 DiI-stained erythrocytes were added into each well and incubated for 24 h. The relative intensity of the images was computed with Image J.
Raw264.7 cells were pre-seeded in 6-well plates incubated for 12 h, and 4×10^6 or 2×10^7 fresh erythrocytes were added to each well and incubated for 12 h. Subsequently, cell supernatant was collected to measure the contents of IL-10, TNF-α and IL-1β with a mouse precoated ELISA kit (LIANKE BIOTECH, CO., LTD).

Western blot analysis: Cellular proteins were extracted by RIPA lysis buffer, and western blot analysis was performed according to the standard protocols. The following antibodies were used for western blotting: NLRP3 (Abcam, 1:1000), β-Actin (Abcam, 1:1000).

Cytotoxicity assays: For the luciferase detection experiment, CT26 cells stably expressing

luciferase were co-cultured with erythrocytes at a ratio of 1:1, each well of 96-well plates contained 100 μL supernatant. The luciferase substrate D-luciferin (potassium salt, 150 μg/mL, Cayman Chemical, USA) was added to a 100 μL/well, and the vitality of the target cells were monitored by Glomax96 Microplate Luminescence Detector (Promega USA). The background luminescence was negligible (<1% of the signal from the wells with only target cells); therefore, the target cell vitality (%) was calculated as:

$$\text{vitality (\%)} = \frac{\text{experimental signal}}{\text{maximal signal}} \times 100\% \quad (1)$$

killing percentage (%) = 100% - vitality (%) (2)

In vivo therapy of tumors with the drug combination: According to the different bleeding methods, we designed two batches of experiments. The design of the first experiment was as follows, mice bearing CT26 tumors were randomly divided into four groups (n=6 of each group). The mice in each group were injected with saline, 2 mg/kg R300, 2 mg/kg zoledronate, the combination of R300 and zoledronate. The second experiment was designed as follows, mice bearing CT26 tumors were randomly divided into four groups (n=5 of each group). The mice in each group were injected with saline, 50 mg/kg CA4P, 1 mg/kg zoledronate, the combination of CA4P and zoledronate. Tumor size was measured every two days using a vernier caliper after the treatment. The tumor volume was calculated according to the following equation: tumor volume = width × width × length / 2.[20]

In vivo analysis of immune cells: The effects of bioorthogonal coordinated nano-aggregation on T cell infiltration at 24 h were researched. Generally, BALB/c mice bearing subcutaneous CT26 tumors (~200 mm^3) were divided into different groups. All these drugs were administered on day 0 and 5 days, then the mice were sacrificed to collect tumors to evaluate the intratumor immune cell infiltration. The evaluation process of tumor immune cell infiltration was as follow. Generally, part of the tumors was enzymatic degraded. Then, ACK Lysis Buffer was used to remove the erythrocytes in tumors. Finally, the cells obtained from tumors were used to detect the $CD3^+$and $CD11c^+$ T cells by flow cytometry assay through using anti-CD11c-FITC antibodie and APC anti-CD3 antibodies. Next, the other part of the

tumors was embedded in OCT and cut into 8 μm slices, followed by staining with FITC anti-CD4 antibody, Alexa Fluor 594 anti-CD8a antibody and APC anti-CD3 antibodies according to the manufacturer's protocols.

Statistical Analysis: All the animal studies were performed after randomization. The GraphPad Prism (v7.0) was used for statistical analysis. The results were presented as mean ± SD with the indicated sample size. Student t-test for two group comparisons and one- or two-way ANOVA were employed for multiple group comparisons. Data were normally distributed and the variance between groups was similar. Statistical significance was determined with 95% (*, $p < 0.05$), 99% (**, $p < 0.01$), 99.9% (***, $p < 0.001$), and 99.99% (****, $p < 0.0001$) confidence intervals. P values > 0.05 represented non-significance (NS).

Data availability: The authors declare that the main data supporting the findings of this study are available within the article and its Extended Information. Additional data are available from the corresponding author upon request.

2. Supplementary Figure

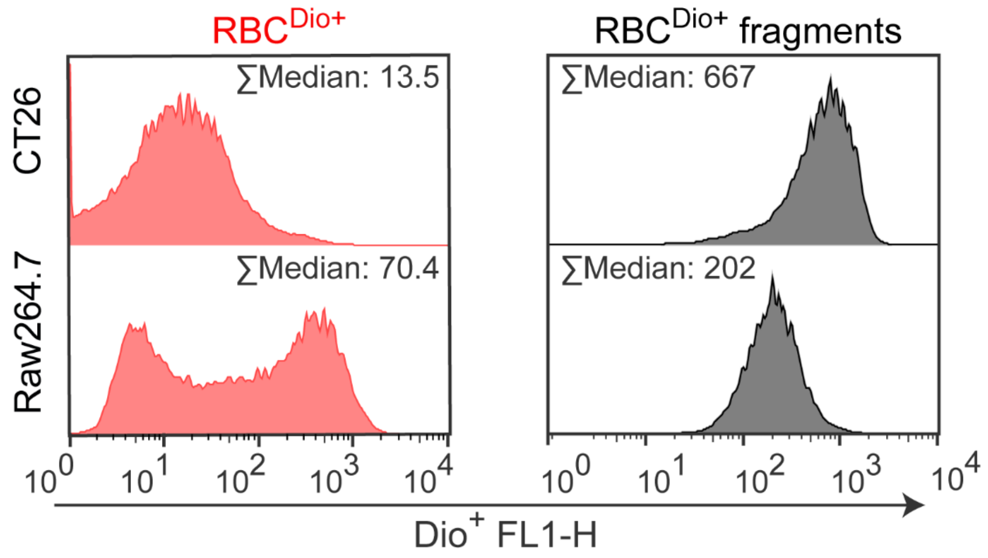

Figure S1. Broken or intact erythrocytes co-incubation with tumor cells as well as macrophages using flow cytometry.

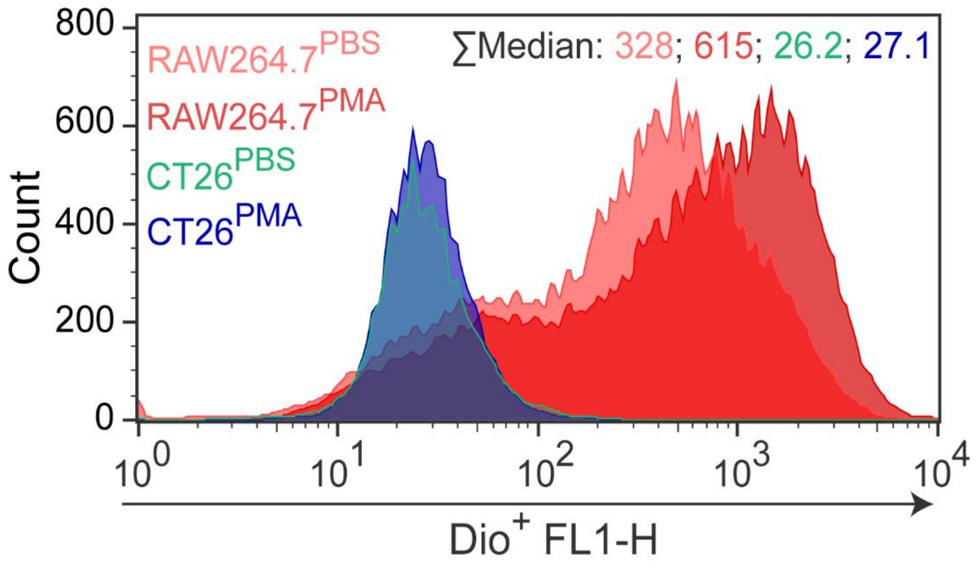

Figure S2. Flow cytometric detection of DiO-stained erythrocytes co-incubation with Raw264.7 macrophages or CT26 tumor cells after treatment with PMA.

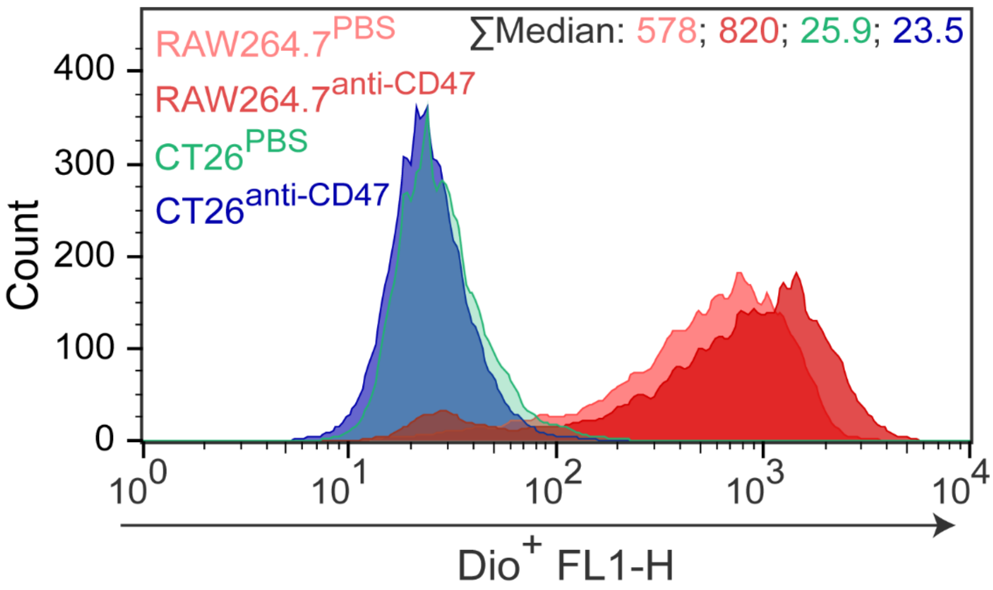

Figure S3. Flow cytometric detection of DiO-stained erythrocytes co-incubation with Raw264.7 macrophages or CT26 tumor cells after treatment with anti-CD47 antibody.

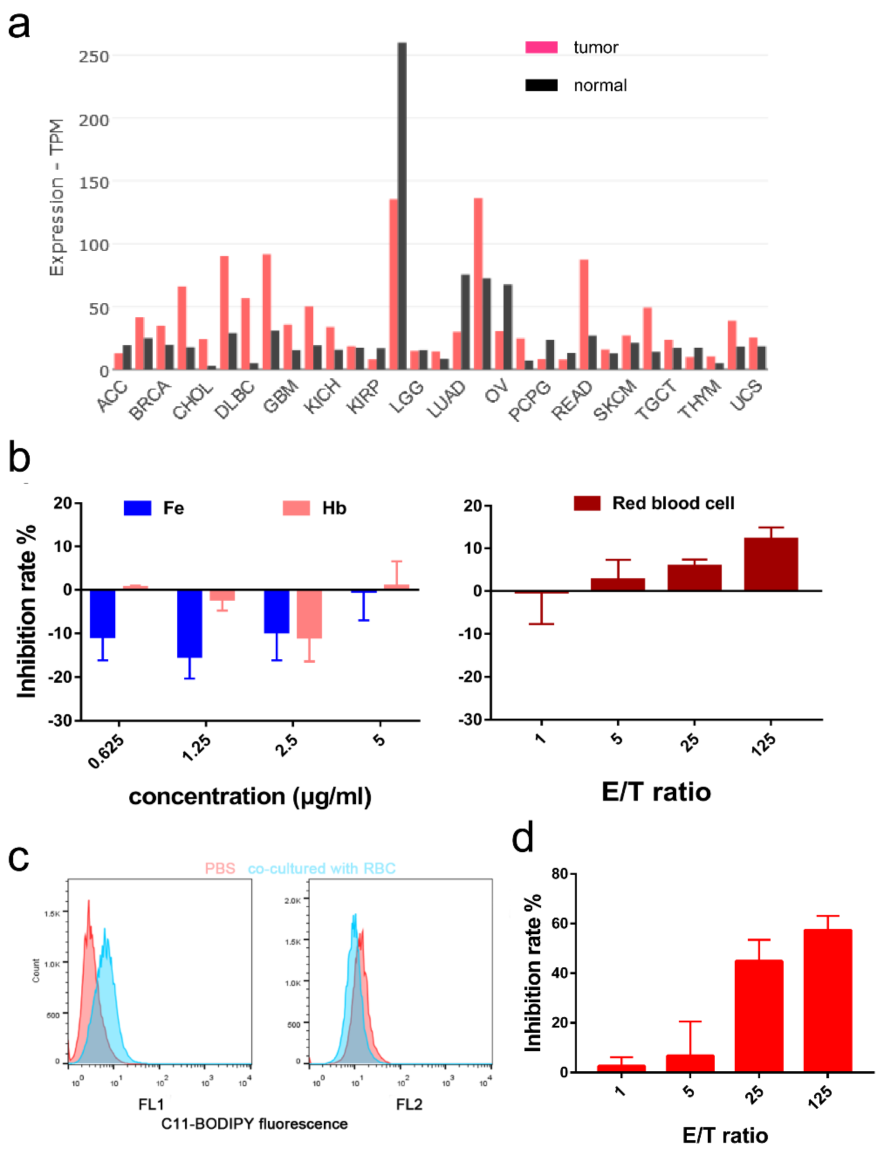

Figure S4. Tumor cell treated with erythrocytes and free iron. a) Transferrin receptor expression in different tumors. **b)** Growth inhibition of CT26 by erythrocytes and free iron. **c)** Erythrocytes produce lipid oxidation. **d)** Growth inhibition of 4T1 by erythrocytes. Data are representative or pooled and expressed as Mean ± SD.

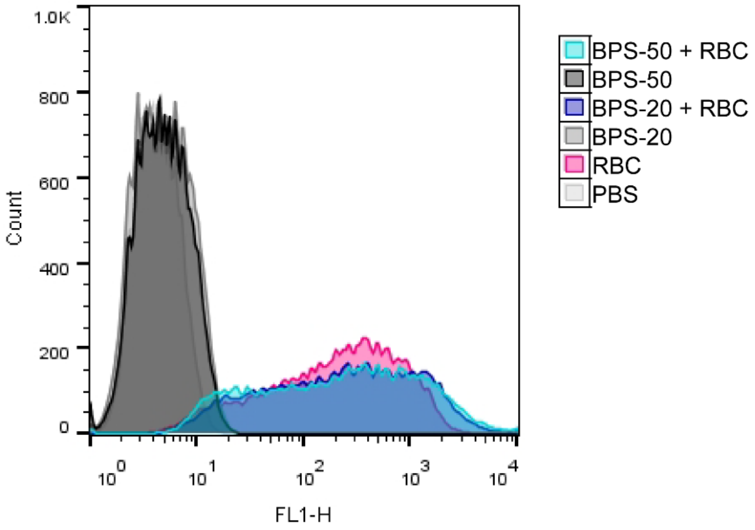

Figure S5. Effect of bisphosphonates on phagocytosis of erythrocytes by macrophages. The data were obtained by flow cytometry.

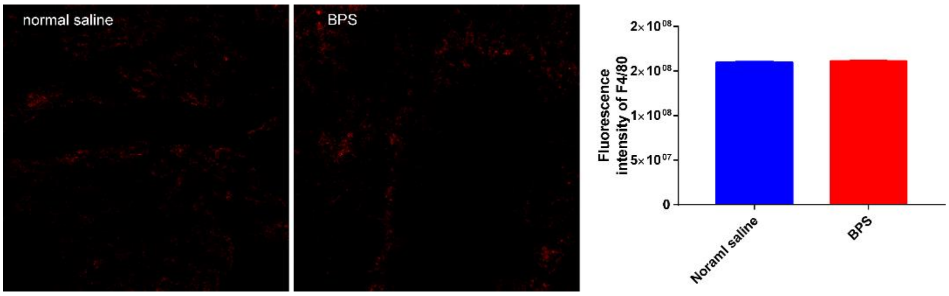

Fig. S6. Quantification of macrophages in tumors. Red fluorescence represents for the F4/80 of macrophage.

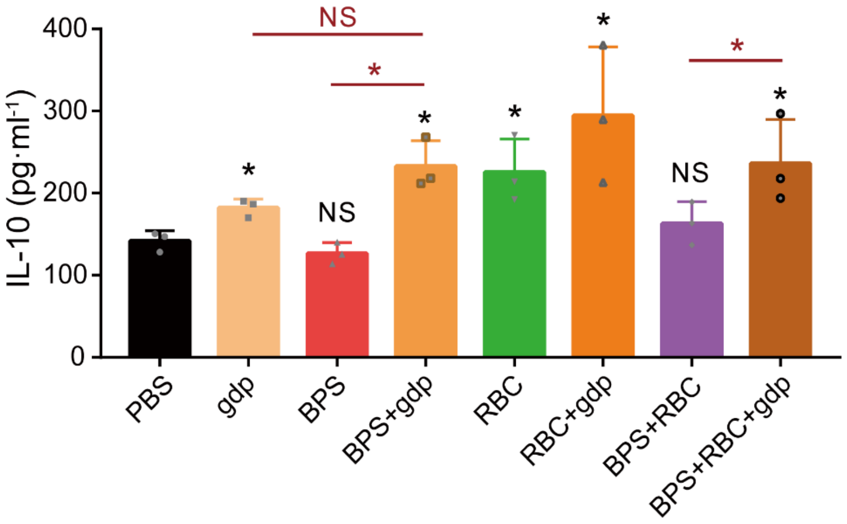

Figure S7. Expression changes of IL-10 after macrophages with erythrocytes and bisphosphonate treatment alone or in combination. gdp: Guanosine 5'-diphosphate disodium salt (gdp), an iron mobilizer.

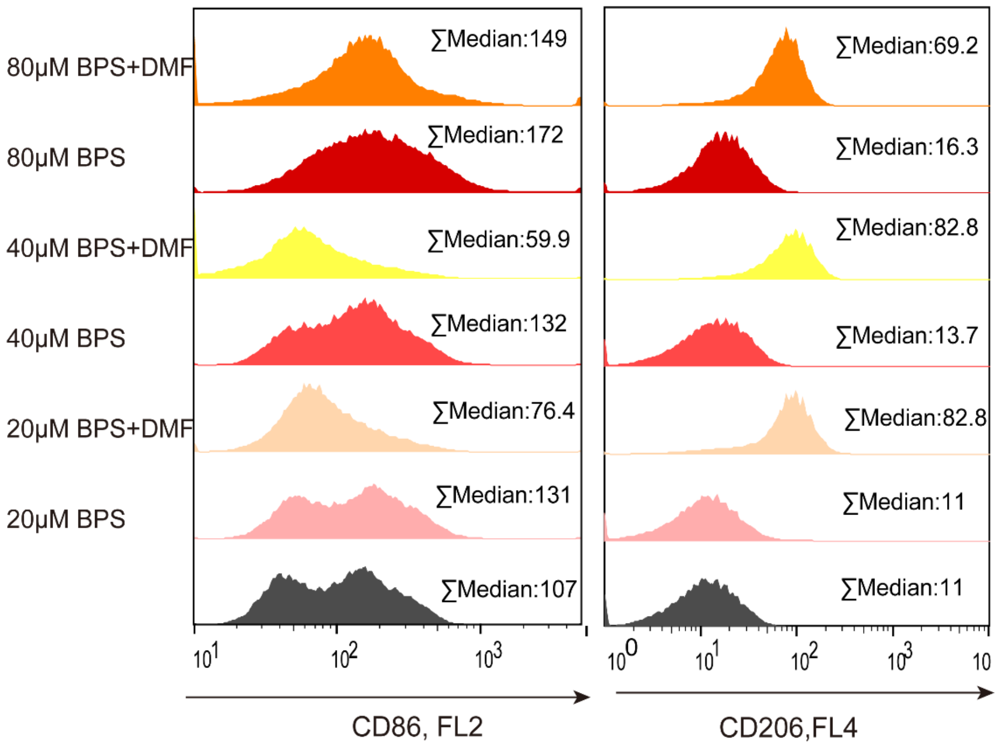

Figure S8. Surface marker expression of macrophages treated with different doses of bisphosphonate and deferoxamine. CD86 and CD206 expression of macrophages were detected by flow cytometry. DMF abbreviation represents deferoxamine.

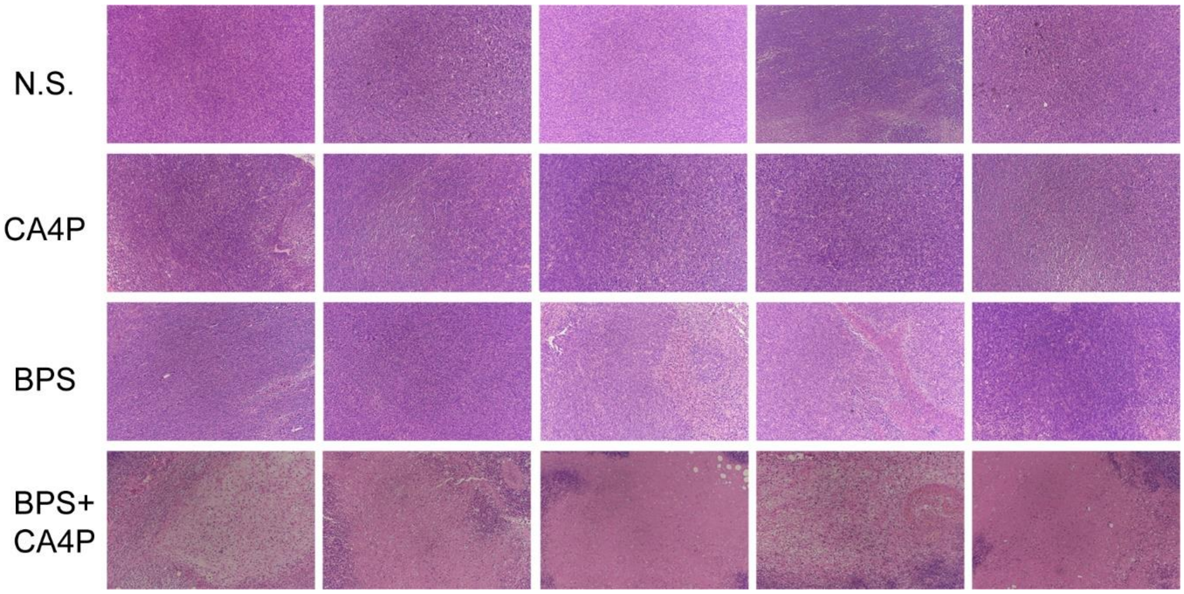

Figure S9. H&E staining of tumor of mice in different treatment groups. CA4P: 50 mg/kg combretastatin A4 disodium phosphate, BPS: 2 mg/kg zoledronate by tail vein injection, CA4P +BPS: the combination of combretastatin A4 disodium phosphate and zoledronate.

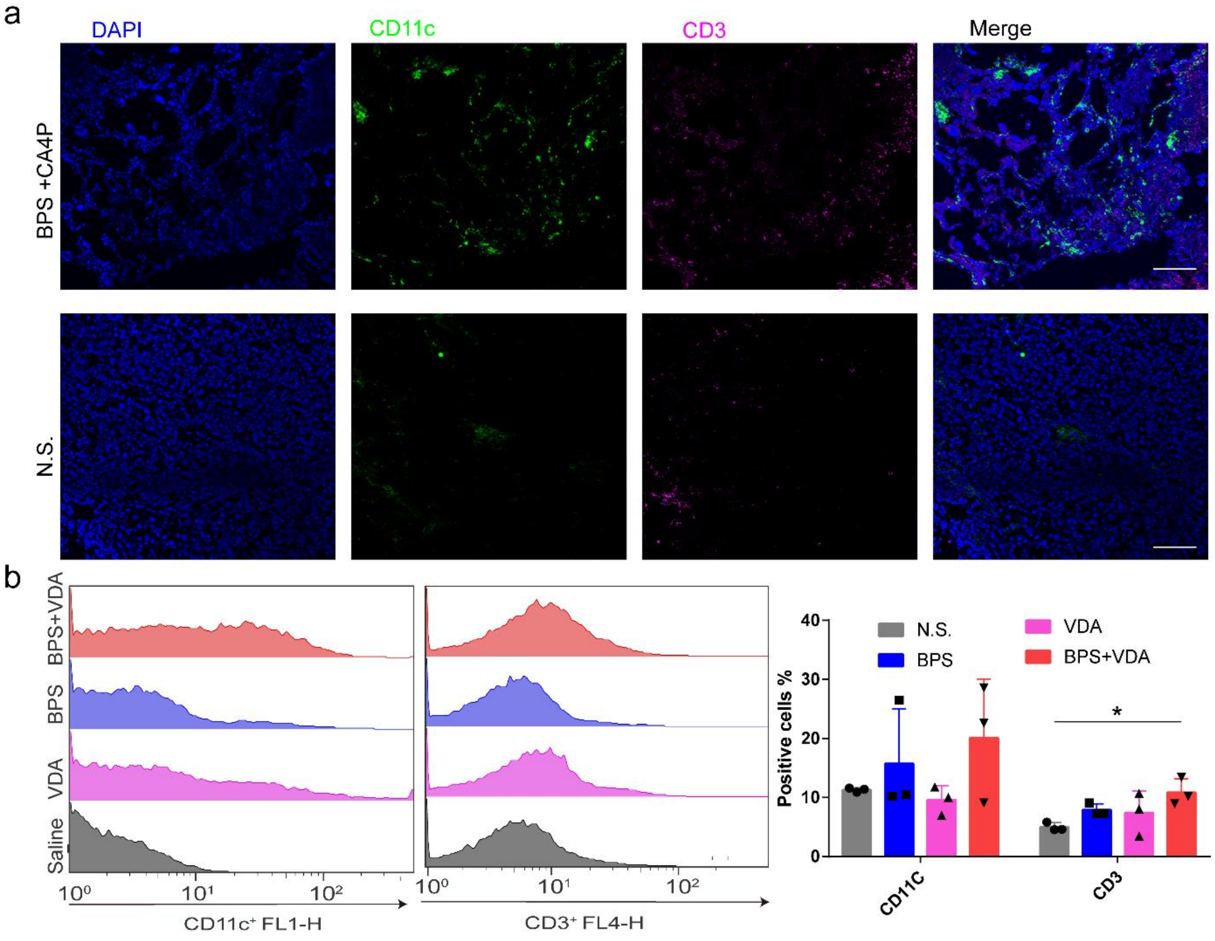

Figure S10. Immunofluorescence and flow cytometric detection of tumor tissues. a)

Tumor tissue was collected and stained after different treatment. Drugs are given at Day 0 by tail vein injection. Scale bars, 100 μm. **b)** Quantification of proportion of $CD3^+$ and $CD11c^+$ cells by flow cytometric detection of tumor tissues. Tumor tissue was collected and stained 5 Days after injection treatment.

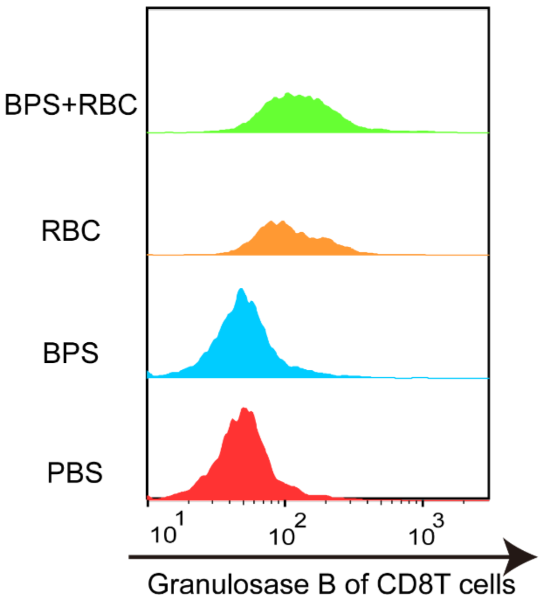

Figure S11. Granzyme B expression by $CD8^+$ T cells following co-incubation with macrophages which treated with erythrocytes and zoledronate. The RBC and BPS abbreviations represent erythrocytes and zoledronate, respectively. The data were obtained by flow cytometry.

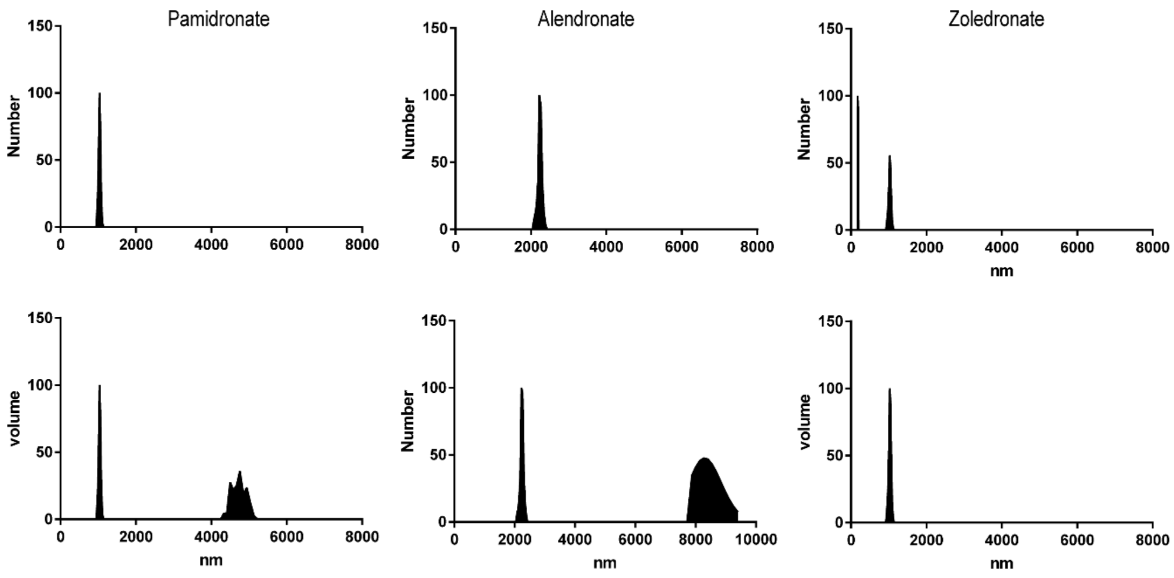

Figure S12. Results of particle size detection after coordination with different bisphosphonates and Fe^{2+}

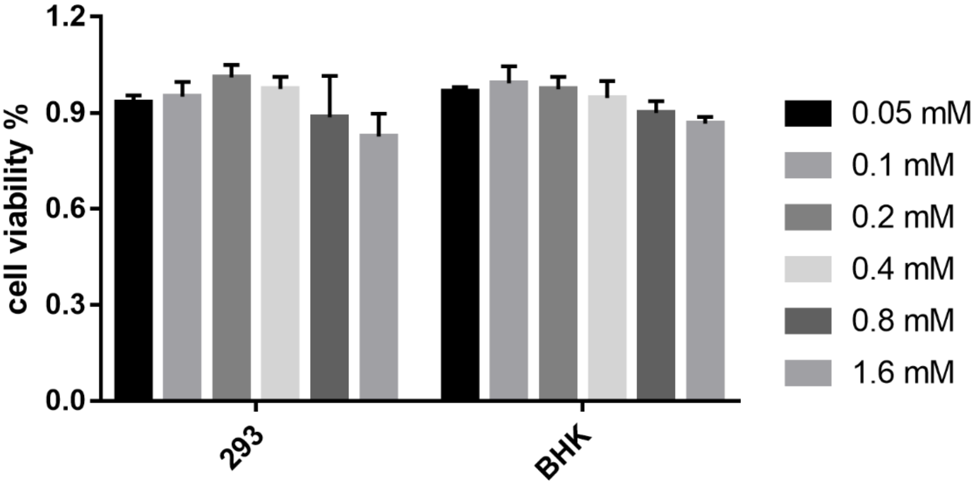

Figure S13. Results of toxicity testing of bisphosphonates on BHK as well as 293.

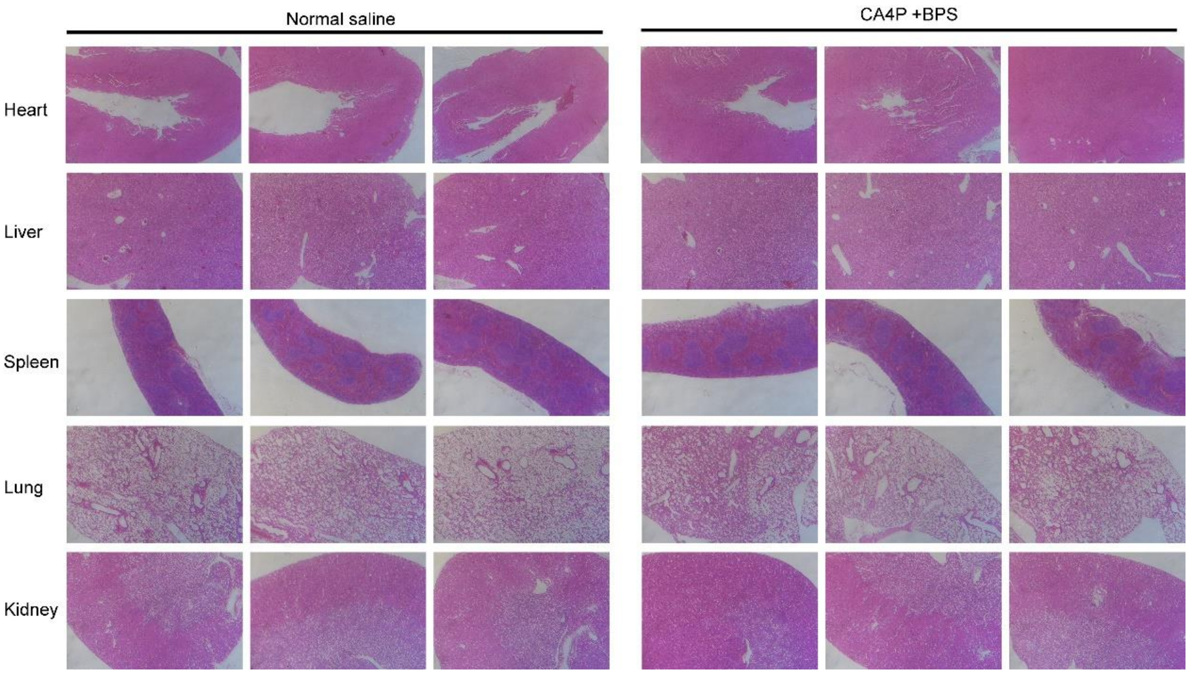

Figure S14. H&E staining of some major organs (heart, liver, spleen, lung, kidney) in mice. CA4P: 50 mg/kg combretastatin A4 disodium phosphate, BPS: 2 mg/kg zoledronate by tail vein injection, CA4P +BPS: the combination of combretastatin A4 disodium phosphate and zoledronate.
